# Novel *Salmonella enterica* Serovar Typhimurium Genotype Levels as Herald of Seasonal Salmonellosis Epidemics 

**DOI:** 10.3201/eid2406.171096

**Published:** 2018-06

**Authors:** Cristina Sotomayor, Qinning Wang, Alicia Arnott, Peter Howard, Kirsty Hope, Ruiting Lan, Vitali Sintchenko

**Affiliations:** Universidad Austral de Chile, Valdivia, Chile (C. Sotomayor);; Centre for Infectious Diseases and Microbiology Public Health, Westmead, New South Wales, Australia (C. Sotomayor, Q. Wang, A. Arnott, P. Howard, V. Sintchenko);; University of Sydney, Sydney, New South Wales, Australia (C. Sotomayor, A. Arnott, V. Sintchenko);; New South Wales Ministry of Health, Sydney (K. Hope);; University of New South Wales, Sydney (R. Lan)

**Keywords:** salmonella, epidemics, molecular epidemiology, genotyping, population diversity, bacteria, enteric infections, *Salmonella enterica* serovar Typhimurium

## Abstract

We examined the population dynamics of *Salmonella enterica* serovar Typhimurium during seasonal salmonellosis epidemics in New South Wales, Australia, during 2009–2016. Of 15,626 isolates, 5%–20% consisted of novel genotypes. Seasons with salmonellosis epidemics were associated with a reduction in novel genotypes in the preceding winter and spring.

Nontyphoidal *Salmonella* spp. cause an estimated 93.8 million salmonellosis infections and 155,000 deaths globally each year (*1*)*.* However, the population dynamics of human salmonellosis remain poorly understood, which can undermine the effective use of public health resources (*2*)*. Salmonella enterica* serovar Typhimurium is a highly diverse serovar and the dominant cause of salmonellosis worldwide (*3**,**4*)*,* experiencing continuous evolution, persistence, and adaptation within different ecologic niches. Whereas the complexities of the clonal structure of *Salmonella* Typhimurium populations have been recognized (*4**–**7*)*,* the effect of temporal change in subtype diversity on disease incidence is not well understood.

Multilocus variable-number tandem-repeat (VNTR) analysis (MLVA) has been used as a high-resolution *Salmonella* typing method amenable to harmonization (*8**,**9*). In our study, we sought to determine if *Salmonella* Typhimurium subtype diversity can be used to predict incidence of human salmonellosis. We examined *Salmonella* Typhimurium isolates recovered during 2009–2016 in the comparatively low prevalence setting of New South Wales (NSW), the most populous state of Australia (Technical Appendix Table).

## The Study

We used MLVA to genotype all *Salmonella* Typhimurium isolates referred to the NSW Enteric Reference Laboratory at the Centre for Infectious Diseases and Microbiology, NSW Health Pathology (Sydney, NSW, Australia), during August 2009–March 2016. We conducted multiplex PCR to amplify VNTRs (STTR9, STTR5, STTR6, STTR10pl and STTR3) and subsequent analyses as described previously (*9**,**10*)*.* We reported MLVA results as a string of 5 numbers representing relevant repeats (*11*). We used *Salmonella* Typhimurium reference strain LT2 (GenBank accession on. NC_003197) as a control throughout and consistently generated the expected MLVA type 4-13-13-10-0211. We defined a cluster as >2 isolates with the same MLVA type collected within 12 weeks (*10*) and used a χ^2^ test to determine the significance of differences observed; we considered a p value of <0.05 significant. We determined population diversity by calculating the Simpson index of diversity (*12*)*,* and population richness using the McIntosh dominance index (*13*). This study was approved by our local Human Research Ethics Committee (LNR/17/WMED/25).

**Figure 1:**
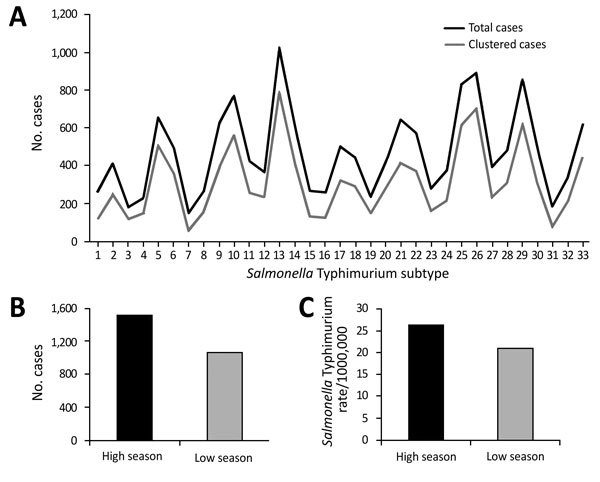
Trends of *Salmonella enterica* serovar Typhimurium notifications and multilocus variable-number tandem-repeat analysis (MLVA) patterns, New South Wales, Australia, 2009–2016. A) Quarterly counts of total cases and cases clustered by MLVA. B) Mean sum of *Salmonella* Typhimurium notifications for summer and autumn quarters for high and low seasons observed (p = 0.01). C) Differences in mean yearly rates of salmonellosis cases reported to the New South Wales Health Department between years corresponding to high and low seasons (p = 0.02). Data source: National Notifiable Diseases Surveillance System (http://www9.health.gov.au/cda/source/).

After excluding duplicate isolates from the same episode of the disease (99% of all cases recorded in NSW), we examined a total of 15,626 human *Salmonella* Typhimurium isolates. We defined seasons as spring (September–November), summer (December–February), autumn (March–May), and winter (June–August). We observed a substantial fluctuation in the number of infections during the study period (Figure 1). The relative contributions of common definitive phage types (DT) and MLVA types to local *Salmonella* Typhimurium activity varied over 8 seasonal peaks. DT135 dominated in 2008 but was subsequently replaced by DT170 as the most common type in 2009 and 2010; an increase in the activity of DT9 occurred in 2014 and 2015. In total, we observed 667 different MLVA types. Six related STM DT170 MLVA types (2-7-6-12-0212; 2-7-7-12-0212; 2-7-6-13-0212; 2-7-6-11-0212; 2-7-6-14-0212 and 2-7-7-11-0212) represented 30% of all isolates. Increases in *Salmonella* Typhimurium cases during summer and autumn months (December–May in the Southern Hemisphere), with 2 cycles of 2009–2011 and 2012–2014, were mirrored by an increase in the number of unique MLVA types detected. The proportions isolates included in clusters also demonstrated expected seasonal fluctuations corresponding to increases in incidence (Figure 1, panel A), although we found no significant change in the number of clusters or their average size during the study period (p<0.05).

The average number of *Salmonella* Typhimurium cases for the 8 summer–autumn seasons investigated during the study period was 1,301. We categorized all summer–autumn seasons as either high (epidemic) or low on the basis of whether case numbers were above or below the average. We confirmed the designations by comparing the seasonal averages calculated for this study with yearly rates of salmonellosis notifications. These 2 approaches congruently assigned summer–autumn seasons of 2010, 2011, 2014, and 2015 as high or epidemic, whereas summer–autumn seasons of 2009, 2012, 2013, and 2016 were classified as low; the difference between case numbers in high and low seasons was significant (p<0.02) (Figure 1, panels B, C). The average annual number of foodborne community outbreaks recorded by the national public health network during high seasons was 63 and during low seasons was 48 (http://www.ozfoodnet.gov.au).

**Figure 2:**
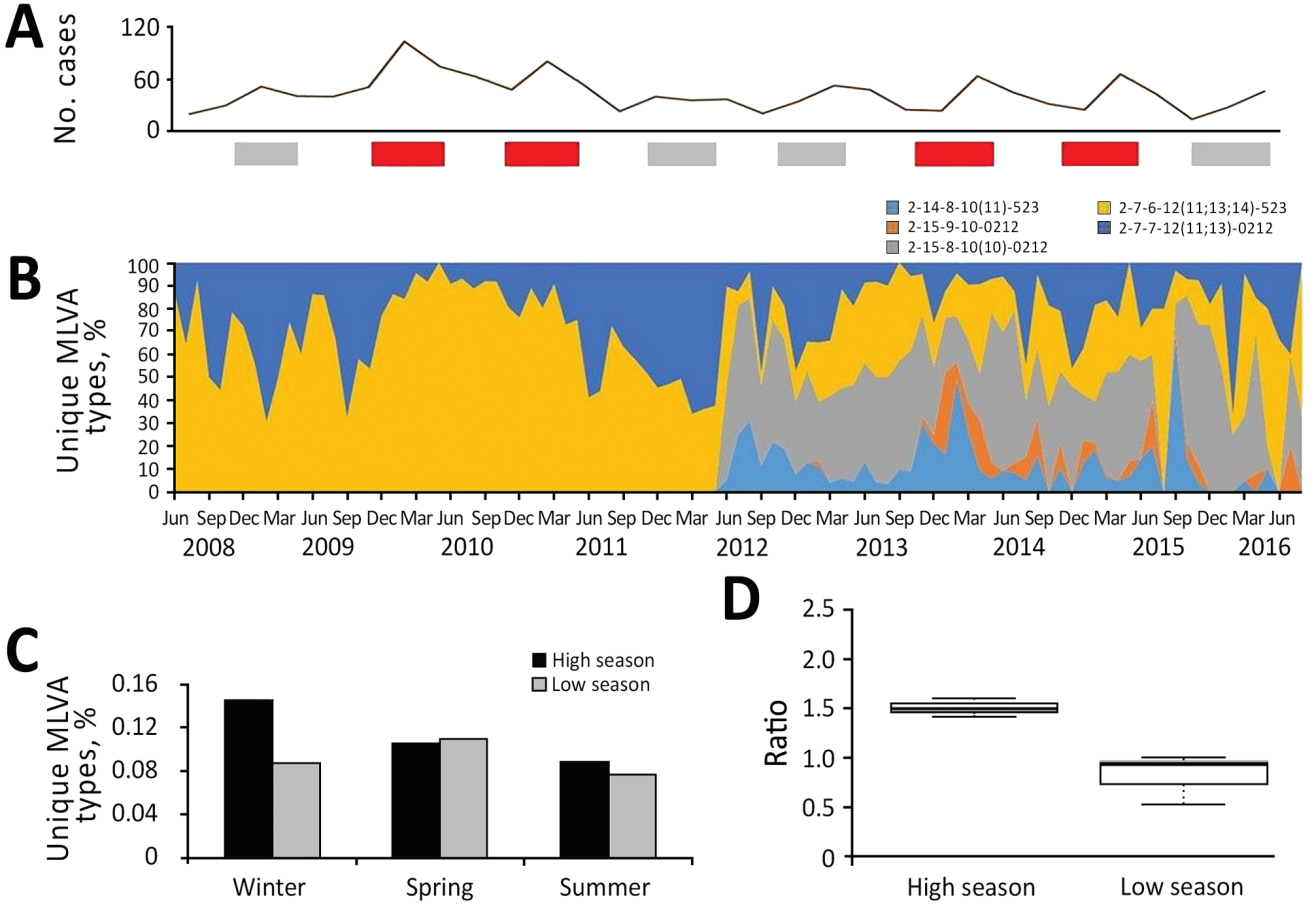
Population dynamics of *Salmonella enterica* serovar Typhimurium MLVA types, New South Wales, Australia, 2009–2016. A) Total number of novel or unique MLVA types. Red bars indicate high season and gray bars low season. B) Temporal dynamics of the most common MLVA types expressed as proportions by type. C) Quarterly counts of novel MLVA types during winter, spring, and summer for high and low seasons (p = 0.05). D) Box plots of the mean ratio of novel MLVA type counts during high and low seasons (p = 0.006). Box top and bottom indicate third and first quartiles, respectively; horizontal lines within boxes indicate medians; whiskers indicate CIs; dotted vertical lines indicate the spread of values in the subgroup. We built box plots with BoxPlotR (http://shiny.chemgrig.org). MLVA, multilocus variable-number tandem-repeat analysis

At the beginning of the study, we considered all MLVA types novel. The ratio of novel, previously unreported MLVA types to all types stabilized within 5 months; after the fifth month, 10%–40% of all MLVA types detected at any given time consisted of novel MLVA types (Figure 2). We detected no changes in the age distribution of human populations affected by dominant types; 41.1% of all infections occurred in those <14 years of age.

The diversity of the *Salmonella* Typhimurium population remained relatively constant over time; the McIntosh dominance index of diversity fluctuated between 0.6 and 0.9 during both high and low seasons (p = 0.478). However, we observed a rapid decrease in the proportions of unique MLVA types from winter to spring (i.e., *U_w_/U_sp_* ratio<1) before epidemic *Salmonella* Typhimurium activity. In contrast, the proportion of unique MLVA types increased from winter to spring preceding low seasons (*U_w_/U_sp_* ratio>1) (Figure 2). This ratio also correlated with incidence of *Salmonella* Typhimurium in NSW over the study period (r = 0.922). Of note, the percentage of unique MLVA types recovered from patients <14 years of age during winter of high/epidemic seasons was also significantly lower than during the winter months of low seasons of salmonellosis (15.1% vs. 28.2%; p<0.001).

Our observations potentially reflect the decrease in *Salmonella* Typhimurium diversity resulting from the reduction of genome variation under selection pressure, which is associated with the emergence of successful unique clones capable of causing epidemics in immunologically naive hosts. However, longer-term monitoring of subtype diversity and disease incidence is warranted to confirm these trends. Although an analogous selection-driven reduction in genetic diversity has been observed in other pathogens, such as influenza virus (*14*)*,* it has not previously been observed in *Salmonella*. Our findings also suggest that the timely recognition of novel *Salmonella* Typhimurium subtypes may be of significance for surveillance and that the conventional diversity indices alone may not be sufficient to detect subtle changes in circulating subtypes. As the estimated ratio of accumulation of MLVA repeats in different loci to single nucleotide polymorphisms is 1:6.9 (*15*), changes in the composition of *Salmonella* Typhimurium subtypes might offer insight into the relevance of population diversity for fluctuations in the incidence of salmonellosis.

## Conclusions

Substantial increases in seasonal epidemics of *Salmonella* Typhimurium can be associated with a reduction in newly identified MLVA types in the preceding winter and spring, reflecting the emergence of successful *Salmonella* Typhimurium clones under selection pressure. The proportion of novel MLVA types in winter and spring may serve as an early warning sign in public health surveillance. These observations add further insights into the epidemiology of *Salmonella* Typhimurium infections in a low-incidence setting. Although they may not be readily applicable to high-incidence *Salmonella* Typhimurium settings with frequent co-infections and different diagnostic or public health practices, the epidemiology of *Salmonella* Typhimurium and public health responses in Australia are similar to those in other industrialized countries, supporting the generalizability of our findings. Prospective monitoring of *Salmonella* Typhimurium population diversity and identifying new MLVA types as reservoirs from which future epidemics might emerge can improve the assessment of risks of seasonal increase in *Salmonella* Typhimurium incidence.

Technical Appendix Additional information about *Salmonella enterica* serovar Typhimurium genotypes.
