# Supplementary material for: Novel Salmonella enterica Serovar Typhimurium Genotype Levels as Herald of Seasonal Salmonellosis Epidemics
Source: Emerg Infect Dis. 2018 Jun;24(6):1079–82. doi: 10.3201/eid2406.171096 (PMC6004855; doi:10.3201/eid2406.171096)
Supplement: Technical Appendix — Additional information about Salmonella enterica serovar Typhimurium genotypes. [file 17-1096-Techapp-s1.pdf]

Article DOI: https://doi.org/10.3201/eid2406.171096

Novel *Salmonella enterica* Serovar Typhimurium Genotype Levels as Herald of Seasonal Salmonellosis Epidemics

Technical Appendix

Technical Appendix Table. Total *Salmonella enterica* serovar Typhimurium isolates and corresponding MLVA profiles, 2009–2016, New South Wales, Australia (n=14,537)

Year	No. isolates (% of total)	No. MLVA profiles(unique/total)
2009	1,565 (10.8)	184/489
2010	2,182 (15.0)	290/436
2011	2,164 (14.9)	198/398
2012	1,600 (11.0)	129/295
2013	1,877 (12.9)	150/360
2014	2,592 (17.8)	166/353
2015	1,878 (12.9)	151/390
2016	679 (4.7)	46/216 (January–March)
